# Potential Roles of Neuroimaging in Motor Neuron Disease

**DOI:** 10.1007/s11910-026-01524-z

**Published:** 2026-09-26

**Authors:** Jan Kassubek, Hans-Peter Müller

**Affiliations:** Department of Neurology, University Hospital Ulm, Oberer Eselsberg 45, D-89081 Ulm, Germany

**Keywords:** Artificial intelligence, Machine learning, Magnetic resonance imaging/MRI, Diffusion tensor imaging/DTI, Volumetry, Arterial spin labeling/ASL, MR spectroscopy, Motor neuron disease, Biomarker, Neurodegeneration

## Abstract

**Purpose of Review:**

The review is intended to summarize and discuss the increasing roles and perspectives of neuroimaging in the clinical and scientific work-up of amyotrophic lateral sclerosis (ALS) and other motor neuron diseases (MND).

**Recent Findings:**

The applications of magnetic resonance imaging (MRI) and positron emission tomography (PET) identify characteristic disease-specific patterns especially for ALS including its clinical subtypes. Guided by neuroanatomical pattern stratification, these MND-associated alterations could be detected by dedicated analysis parameters from MRI, including T1-weighted structural imaging and microstructural diffusion tensor imaging, in combination with additional MR techniques, to act as diagnostic and monitoring biomarkers. Imaging in MND increasingly makes use of machine learning-based analysis methods.

**Summary:**

The conceptualization of (neuro-)imaging in ALS and other MND encompasses multiparametric technical approaches, also of regions outside the central nervous system, together with aspects of disease-specific pathophysiology which will further advance the field in future applications.

## Introduction

Amyotrophic lateral sclerosis (ALS), as the most common adult-onset motor neuron disease (MND), has a core phenotype of progressive degeneration of upper and lower motor neurons [1, 2] and is a primarily clinical diagnosis based on standardized international consensus criteria like the Gold Coast criteria [3, 4]. Increasing evidence has been gathered that ALS is a multisystem neurodegenerative disorder involving the accumulation and spread of misfolded proteins, i.e. TDP-43 [5, 6]. In sporadic ALS, TDP-43 pathology primarily appears to stereotypically propagate along anatomically connected neuronal networks, with a sequential spread from the motor cortex to the brainstem and spinal cord, followed by frontal, parietal, and anteromedial temporal regions [5, 7]. Advances in genetic discoveries, especially of the *C9orf72* repeat expansion, have moved forward the understanding of ALS pathogenesis including gene-targeted therapies [8]. Clinically, ALS presents with considerable heterogeneity with subphenotypes that deviate from the “classical” involvement of upper and lower motor neurons, including primary lateral sclerosis (PLS), bulbar-onset ALS/progressive bulbar palsy (PBP), progressive muscular atrophy (PMA), and flail arm syndrome (FAS) which differ in motor neuron involvement, site of onset, disease progression, and prognosis [9].

**Fig. 1:**
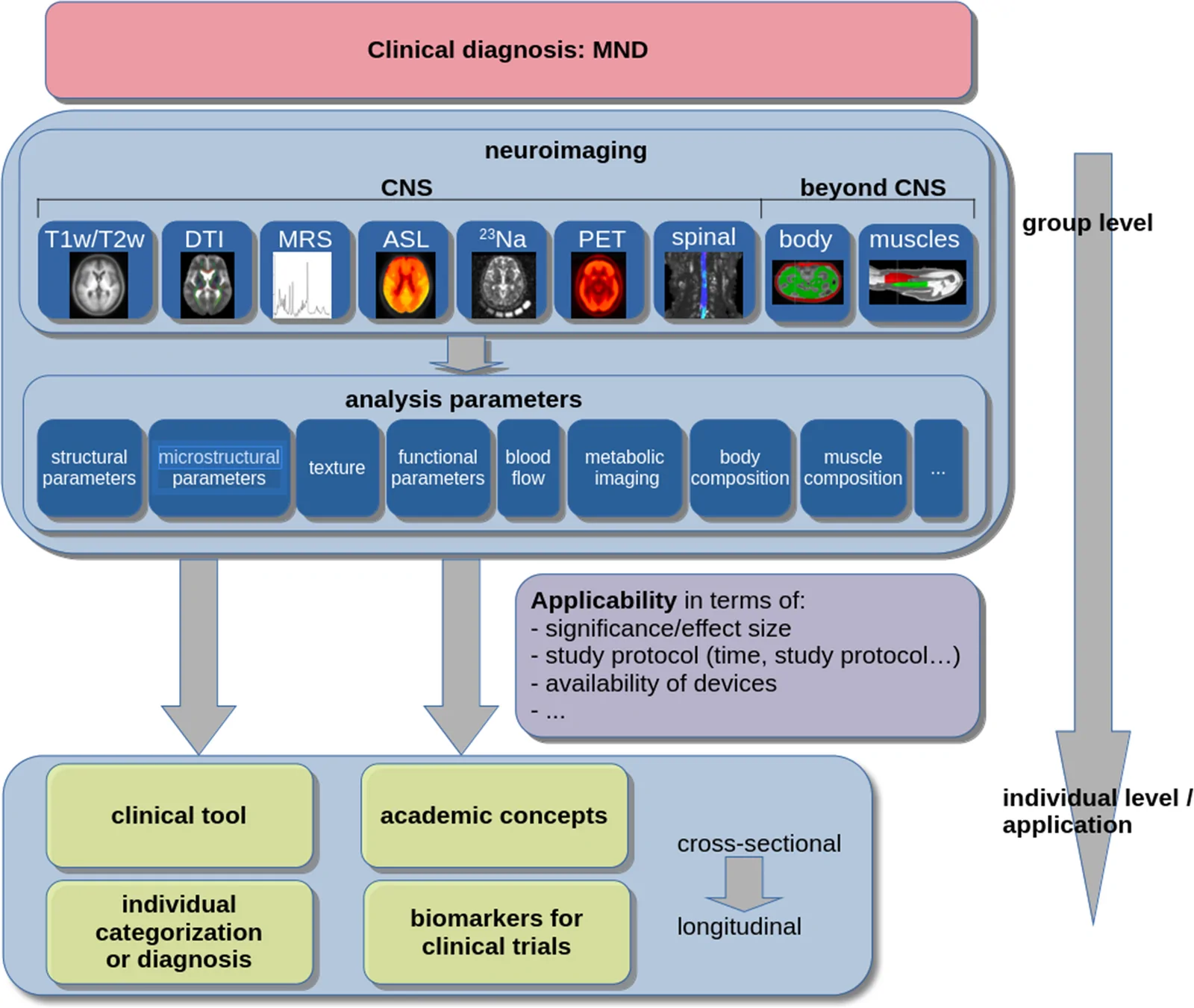
Neuroimaging in motor neuron diseases (MND). MND patients are scanned by different neuroimaging contrasts. Resulting analysis parameters could undergo were tested on their applicability to act for individual categorization or diagnosis or to obtain biomarkers for clinical trials. T1w – T1-weighted; T2w – T2-weighted; DTI – diffusion tensor imaging; MRS – magnetic resonance spectroscopy; ASL – arterial spin labelling; PET – positron emission tomography; CNS – central nervous system

The clinical diagnosis is supported not only by electrophysiology and fluid biomarkers from serum and cerebrospinal fluid, but also neuroimaging [10], i.e., imaging mainly with magnetic resonance imaging (MRI) plays an important role in the diagnostic workup of ALS and in excluding alternative conditions [11]. The potential of multiparametric MRI in MND both from a clinico-diagnostic and from a neuroscientific perspective has been widely acknowledged for decades [12]. Beyond clinical progress, advanced structural, microstructural, and functional MRI techniques have emerged as valuable tools for investigating ALS pathology in vivo, with potential applications in phenotype stratification and monitoring disease progression [13, 14]. By combined technical approaches, longitudinal propagation patterns, neuropathological staging, and correlates of disease burden including adaptive changes can be addressed [15, 16] (Fig. 1). In a multimodal approach, PET and PET MRI hybrid imaging, respectively, have additional value by multi-tracer and multi-isotope studies, potentially combined with the high spatial information provided by MRI to improve signal separation [17].

## Microstructural Alterations, Assessed by Diffusion Tensor Imaging

Diffusion tensor imaging (DTI) enables the assessment of axonal damage and myelin degeneration through diffusion metrics, particularly fractional anisotropy (FA), and has been successfully used to map white matter (WM) pathology and disease propagation in ALS and its variants [10, 12, 18, 19, 20]. Analyses can be performed using unbiased voxelwise methods like whole-brain-based spatial statistics (WBSS) [21] and tract-based spatial statistics (TBSS) [22], or hypothesis-driven approaches including tract-of-interest (TOI) mapping and tractwise FA statistics (TFAS) [23, 24]. TOI-based analyses have demonstrated sequential involvement of ALS-related WM tracts in both cross-sectional and longitudinal studies, including an in vivo transfer of the TDP43-based neuropathological disease staging [23, 25]. Advanced diffusion techniques like neurite orientation dispersion and density imaging (NODDI) provide more detailed diffusivity profiles including crossing fibers and specifically identified regional dendritic changes [26, 27]. Overall, DTI-derived metrics constitute highly valuable imaging biomarkers for microstructural changes in ALS and may contribute to disease staging and progression monitoring [28, 29]. Longitudinal studies by unbiased voxelwise DTI analyses in ALS have demonstrated significant regional FA reductions, mainly within the corticospinal tract (CST) and frontal regions [25, 30, 14, 29]. Meta-analyses further showed that neurodegeneration extends beyond the motor system into extra-motor regions, supporting the concept of ALS as a multisystem WM disorder [31].

Multicenter DTI studies for the analysis of larger patient cohorts are challenging e.g. due to scanner-related variability. For FA, harmonization techniques have been employed to reduce inter-site variability for the pooling of data acquired with different protocols [32]. Using these approaches, international multicenter studies were able to confirm ALS-related WM alterations in standardized DTI protocols of large patient cohorts [33], demonstrating the potential to monitor ALS-related changes also in a setting of therapy evaluations.

Hypothesis-driven DTI studies in ALS have been performed based on the neuropathological corticoefferent propagation model of TDP-43 pathology [5, 34]. This staging model has been translated to in vivo MRI using cross-sectional and longitudinal TOI-based analyses of WM pathways [23, 25], with the highest frequency of abnormalities in the CST (stage 1), followed by corticorubral/-pontine tracts (stage 2), corticostriatal pathway (stage 3), and hippocampal paths (perforant path, stage 4). In these studies, diffusion metric changes correlated with the proposed four-stage disease model and disease severity [25, 35]. A systematic meta-analysis confirmed a pattern of FA alterations corresponding to the proposed neuropathological staging of ALS [36], as in vivo support for the TDP-43-related propagation model of ALS pathology.

In that context, it is of note that ALS exhibits considerable clinical heterogeneity, including restricted phenotypes such as LMND/PMA, PLS, PBP, and FAS [37, 38]. Neuroimaging has been applied to patient groups with these clinical variants in order to evaluate common factors and differences. A multiparametric MRI study in PLS, using T1-weighted and diffusion MRI data, provided evidence that cerebral areas showing the most significant anatomical associations between atrophy and mitochondrial density (i.e., precentral gyrus, cerebellum, frontotemporal regions) are pathognomonic brain regions of PLS [39]. Specifically, DTI studies have demonstrated characteristic WM abnormalities in all these endophenotypes corresponding to the DTI-based corticoefferent staging model, including PMA despite no overt first motor neuron involvement [40, 41]. In PLS also, tract-specific analyses showed the same propagation pattern as `classical` ALS [42, 43]. Similarly, microstructural involvement patterns corresponding to the ALS staging model were demonstrated in PBP [44] and FAS [45], respectively, supporting their classification as ALS variants. As a consequence, TOI-based FA mapping has substantially contributed to the recognition in vivo that the above-named restricted phenotypes share the same underlying WM pathology as classical ALS [10].

In the sense of a stratification by genotype, neuroimaging has identified distinct structural and microstructural abnormalities in genetic ALS, particularly in patients carrying the *C9orf72* hexanucleotide repeat expansion, which is associated with a more aggressive disease course and cognitive impairment: MRI studies demonstrated reduced WM integrity extending beyond the motor system [46]. DTI in these patients also revealed FA reductions along the tract systems corresponding to the corticoefferent neuropathological spreading pattern [47], consistent with histopathological findings [7]. Additional frontal callosal involvement suggests an overlap between motor neuron degeneration and frontotemporal pathology in *C9orf72*-associated ALS [48]. Multiparametric MRI further expanded this neuroimaging signature of C9orf72-associated ALS with a distinct pattern of widespread WM and grey matter (GM) alterations which correlate with the clinical phenotype including the cognitive dysfunction profile [49].

Longitudinal imaging studies of presymptomatic mutation carriers provide valuable insights into the earliest stages of genetic ALS [50]. Presymptomatic carriers of *SOD1* mutations exhibit a specifically distinct imaging-based pattern of cortical and subcortical involvement [51, 52]. In asymptomatic *C9orf72* carriers, structural and functional thalamic abnormalities, as well as selective hippocampal alterations, can be detected as potential correlates of the disease process before overt cortical degeneration [52]. Distinctive atrophy patterns were found years before symptom onset on presymptomatic scans of phenoconverters with a *C9orf72* mutation, and the analysis of longitudinal similarity measures may serve as a promising imaging biomarker for identifying those at risk of ALS (or ALS-FTD) [53]. These findings highlight the potential of neuroimaging biomarkers for genotype-specific stratification and development of even presymptomatic imaging-based signatures in MND.

### Further DWI-Based Analysis Techniques

Diffusion kurtosis imaging (DKI) extends DTI, allowing a more accurate characterization of complex tissue microstructure and non-Gaussian diffusion [54]. In ALS, DKI may improve early detection and characterization of WM pathology within multiparametric imaging frameworks [55], also in relationship with the clinical phenotype like bulbar onset [56, 57].

It has been proposed that glymphatic system dysfunction may contribute to the pathophysiology of ALS [58], and MRI-based markers of perivascular diffusion (e.g., diffusion along perivascular spaces) have demonstrated altered glymphatic function in ALS patients compared to controls. Recent study added evidence that putative glymphatic dysfunction co-occurs with extracellular fluid alterations, WM microstructural changes, and clinical impairment in ALS [59]. In addition, the choroid plexus, a key structure in glymphatic function, is increasingly recognized to demonstrate structural alterations, i.e., enlargement, in association with ALS; here, Ma and colleagues reported progressive choroid plexus enlargement that emerges early in ALS and suggested these findings as a promising neuroimaging marker in ALS patients for monitoring disease progression and neuroinflammation-related changes [60]. Additional studies in larger patient samples are in progress for the assessment of the glymphatic system in ALS.

## Metabolism Alterations, Detected by Positron Emission Tomography

PET allows not only for the quantification of regional cerebral metabolism, characteristically altered in ALS, but also enables molecular-level quantification of neuroinflammation, neurotransmission, and protein and receptor density, making it a valuable tool in ALS research [61]. ^18^F-FDG-PET studies have shown stage-dependent metabolic alterations in the motor cortex and associations between prefrontal/limbic hypometabolism and behavioral symptoms such as apathy [62]. In presymptomatic *C9orf72* carriers, PET with MRI-based partial volume correction can detect early metabolic changes [63], while precentral hypometabolism may distinguish *SOD1*-associated ALS from sporadic forms [64]. FDG-PET imaging revealed similar widespread hypometabolism in PMA, as in ALS, whereas PLS showed a more focal motor cortical pattern of hypometabolism: despite the clinical differences, PMA and ALS showed similar FDG-PET metabolic patterns, whereas PLS exhibited a more restricted cortical signature in this retrospective study [65]. Changes of connectivity of motor and cognitive areas with temporal and cerebellar regions among different King’s stages [66] might reflect the spread of TDP-43 proteinopathy or a compensatory mechanism, respectively [67].

The integration of PET with MRI offers complementary structural and functional information, with the potential to establish a comprehensive `one-stop` imaging approach for biomarker development in motor neuron disease [68, 69, 70]. Integrated PET/MRI further allows assessment of small structures such as the brainstem and cervical spinal cord, revealing metabolic changes potentially related to microglial activation [71]. That way, multimodal imaging approaches combining PET and MRI improve disease characterization, enable patient stratification by progression rate, and support biomarker development for clinical trials and personalized therapy [17, 72]. PET/MRI is therefore emerging as a promising platform for standardized, biologically grounded assessment of ALS, although further validation is required for routine clinical use.

## Macroscopic Alterations, Detected by Structural MRI

### MND-Related CNS Abnormalities in Structural/Morphological Imaging

Findings in standard acquisitions in routine clinical protocols, such as CST hyperintensities on T2-weighted/FLAIR images or a T2-hypointense precentral gyrus rim or precentral gyrus atrophy, have low sensitivity and specificity and are not recommended for diagnostic use [10], although additional analyses suggested that e.g. ALS patients with CST hyperintensities may represent a distinct subgroup [73]. Cortical atrophy with a focus in the motor cortex has been frequently reported, along with frontotemporal involvement in ALS and ALS-FTD, including multiple structures which are involved in cognitive/memory processing [74]. Unbiased group-level MRI studies using voxel-based and intensity-based approaches have also demonstrated widespread involvement in ALS, in association with the clinical phenotype [75]. ALS-associated degeneration of the corpus callosum as the largest WM structure of the brain, confirmed by post mortem studies [76], was repeatedly demonstrated in vivo by computational neuroimaging as part of the WM pathology signature in ALS. Most of the MRI studies of the corpus callosum alterations in ALS, with the most prominent findings localized to motor-related areas corresponding to segment III connected to the primary motor cortex, were performed by use of DTI [77, 47, 78] which is specifically prone to map the microstructural alterations of this fiber structure. These corpus callosum alterations have been identified both in ALS and in PLS with phenotype-specific involvement of callosal subregions by use of macrostructural texture analysis of T1-weighted MRI [47, 70]. Beyond this specific application to region-based analyses, macrostructural whole-brain-based texture analysis of T1-weighted MRI enables quantitative voxelwise analysis of WM alterations in ALS using gray-level co-occurrence matrix (GLCM) methods [79, 80]. Studies have shown significant voxel-wise differences between ALS patients and controls along the CST, achieving high classification accuracy and supporting a link between texture features and CST degeneration [81]. These texture measures encompass associations with survival and disease progression, distinguishing slow and fast progressors [82, 83]. Multimodal longitudinal brain changes in presymptomatic *C9orf72* disease were quantified [84], showing faster atrophy in carriers, localized in putamen, insula and cerebellar regions and mean diffusivity increase mainly in uncinate and thalamo-cortical tracts.

### Hypothalamic Atrophy

One finding based on neuroimaging data deserves specific attention. Despite its predominant motor phenotype, ALS is conceptualized as a multisystem neurodegenerative disorder with non-motor features such as a hypermetabolic state that may precede and accompany symptom onset [85]. A recent multiparametric neuroimaging study has investigated functional changes of the neurophysiology of food intake processing/appetite in ALS [86]. In that context, regional atrophy of the hypothalamus, as a core CNS structure modulating appetite and metabolism, has consistently been demonstrated by MRI studies [87, 88, 89, 90, 91], including analyses in specific phenotypes like PLS (Kassubek et al., 2025) and even in presymptomatic mutation carriers [87]. These structural changes are considered to be associated with altered body mass index and changes in visceral and subcutaneous fat composition [92]. The hypothalamic atrophy has been shown to correlate with lower BMI [90], and a multiparametric MRI study demonstrated multifold strucural and functional alterations of the hypothalamic connections in ALS patients [93], in accordance with a previous translational neuroimaging study [94]. However, potential clinical or even therapeutic implications of the hypothalamic alterations in volume and connectivity with respect to prognostic stratification or evaluating intervention effects remain an open issue yet.

### ^23^Na Imaging

^23^Na MRI measures total tissue sodium concentration in vivo, a marker of cellular integrity and ionic homeostasis. In ALS, elevated sodium levels have been detected in GM and WM motor areas, including the precentral gyri, corticospinal tract, and corpus callosum, suggesting early metabolic and structural dysfunction [95, 96]. Current data suggest that total sodium concentration increase assessed at the individual level by ^23^Na MRI may be a useful marker of the clinical heterogeneity of ALS patients [97].

### Arterial Spin Labeling (ASL)

In contrast to traditional perfusion MRI performed using gadolinium-based techniques such as dynamic contrast-enhanced and dynamic susceptibility contrast imaging, arterial spin labeling (ASL) provides a non-contrast measure of cerebral blood flow and has potential clinical relevance in neurological disorders [98]. After early ASL studies in ALS reported correlations between GM perfusion and disease severity, ASL was suggested as a possible disease neuroimaging marker [99]. However, later studies have demonstrated variable group-level effects in ALS, limiting its current clinical utility [100, 101]. A consistent finding across these ASL studies was hypoperfusion in both motor regions, correlated with functional impairment, and non-motor regions, particularly in the frontotemporal cortex [74].

### Magnetic Resonance Spectroscopy (MRS)

Magnetic resonance spectroscopy has revealed metabolic disruptions in ALS, including reduced N-acetylaspartate and elevated choline levels, most pronounced in the motor cortex and CST with extension to extra-motor regions [74]. MRS also improves diagnostic accuracy when combined with DTI and reveals subgroup-specific and regionally distinct metabolic alterations, including in the hippocampus and supplementary motor area [102]. MRS with ^1^H and ^31^P imaging at ultrahigh field was able to differentiate patterns of altered brain metabolism in patients with *C9orf72* mutation, patients without this mutation, and asymptomatic mutation carriers [103].

### Spinal Cord MRI

From the clinico-diagnostic perspective, spinal cord MRI is primarily used to differentiate MND from their mimics like degenerative cervical myelopathy [104]. With respect to advanced spinal cord MRI techniques [105], DTI is the most valuable so far, although further standardization and validation are required for routine clinical use [106, 107]. Quantitative brainstem and spinal cord MRI, including T2-weighted and DTI measures, can help predict motor decline, respiratory dysfunction, and ventilation needs in ALS [108, 109]. Multimodal spinal cord MRI combining complementary metrics improves diagnostic performance, achieving high accuracy compared to single-modality approaches [110]. However, larger standardized studies with robust clinical correlation and automated analysis pipelines are needed to confirm its clinical value in ALS.

## Imaging of Body Parts Beyond the Central Nervous System

As a neurodegenerative disease, the diagnostic and research focus of imaging in MND has traditionally been targeted on the central nervous system (CNS), i.e., the brain and the spinal cord. However, MRI of anatomical regions outside of the CNS has proven potential to considerably contribute to the pathophysiological understanding and disease monitoring of ALS. One major field is muscle imaging which can help to visualize changes in the muscles, such as muscle atrophy or changes in muscle tissue, in order to define and quantify patterns of involvement in order to better understand the progression of the disease [111]. For a large variety of non-invasive quantitative muscle imaging techniques, their potential clinical utility have been successfully demonstrated [112], and also from an academic view, muscle MRI can contribute to analyze clinical spreading patterns at the downstream/muscular level [113]. The role of recent technical developments remains to be explored, including multiple-point diffusion-weighted stimulated echo imaging to measure fasciculations [114] and motor unit MRI [115]. As one specific muscle, the tongue has been investigated by MRI, and recently a multi-contrast T1-weighted/T2-weighted MRI protocol for the measurement of the volumes of key tongue muscles in patients with MND has been proposed [116]. In addition, tongue atrophy has been quantified by AI-assisted volume measurements in T1-weighted MRI data both in the PBP variant of ALS [117] and also in spinobulbar muscular atrophy [118]. Another domain of imaging beyond the CNS in MND is mapping of bodyfat/body composition as a structural correlate of ALS-associated bodyweight loss (hypermetabolic syndrome) [119]. An automatic whole-body-based MRI analysis technique for separate determination of the subcutaneous and visceral fat volumes in selected body regions demonstrated that ALS patients had more visceral and less subcutaneous fat and that higher subcutaneous fat predicted longer survival [120]. By integrating body composition analysis - by MRI, but also by DEXA [121] - into future multimodal approaches, clinicians will probably use these techniques for patient prognostication and to tailor nutrient plans. A detailed review of imaging beyond the CNS in MND has been recently provided [122].

## Conclusions

### The Future of Imaging in ALS – Multiparametric Imaging

Overall, T1- and T2-weighted MRI texture and structural analyses show promise as group-level biomarkers of ALS pathology, but larger studies are needed for clinical translation and individual diagnostic use. These approaches may become valuable components for future multimodal and AI-based imaging frameworks. A multiparametric MRI approach combining structural, microstructural, and functional measures is to be proposed to improve classification in ALS [123, 124]. Such models integrate on the one hand a variety of technical imaging approaches, mainly with data from techniques like T1-weighted imaging and DTI, but also other techniques (which still require standardization initiatives). On the other hand, based on the existing knowledge from neuropathological studies and – in the light of the lack of autopsies in current times – neuroimaging itself, specific neuroanatomical structures known to be key elements of the disease process in ALS can be specifically investigated. A composite score incorporating changes in such regions, including but not limited to the corticospinal tract, corpus callosum (segment III), motor and frontotemporal cortices, and the hypothalamus, might enhance the diagnostic value.

Methodologically, combining GM and WM–focused metrics appears most promising, also in the characterisation of neuroimaging-based fingerprints of specific ALS phenotypes or genotypes [49]. Large multimodal studies have demonstrated progressive cortical and subcortical atrophy, ventricular enlargement, and WM degeneration in motor and frontotemporal networks, supporting the value of longitudinal multiparametric imaging in ALS [125]. An advanced approach with the combination of GM density and WM microstructural integrity mapping with contrastive trajectory inference was able to identify three ALS subtrajectories that displayed distinct alterations in the motor, limbic system, and widespread cortical and subcortical changes, with differences in the clinical symptoms [126]. Multimodal and stage-specific neuroimaging integration, used to improve diagnostic accuracy and illuminate distinct disease phases, will support ALS patient stratification by progression rate or molecular subtype, enrichment of clinical trial cohorts, and the development of surrogate endpoints [127].

### The Future of Imaging in ALS – Artificial Intelligence

Artificial intelligence (AI) and machine learning (ML) are increasingly used in neurology to support diagnosis, prognosis, and disease monitoring [128]. Deep learning for neuroimaging in neurodegeneration can improve early diagnosis and disease monitoring, although its implementation faces challenges in the basic real-world data, including but not limited to inter-site and inter-scanner variability and class imbalances in medical datasets [129]. By ML applications to ALS, observer bias is reduced by enabling automated, quantitative analysis and integrating multimodal data such as T1-weighted imaging and DTI [130, 131]. However, an evaluation with hypothesis-guided information about the specific neuroanatomical key elements of the disease process will additionally help to increase the quality of the analysis results.

In ALS, ML methods—including support vector machines, random forests, clustering approaches, and deep learning—have been used to classify patients, identify subtypes, and predict disease progression with promising accuracy [24, 123, 131, 132, 133]. Multimodal AI can capture patterns consistent with disease spreading and clinical symptoms. Many of the more recent studies presented in this review have used AI-based algorithms. As another example of AI-based optimisation of already primarily unbiased analysis approaches, a recent longitudinal MRI study combined deformation-based morphometry (DBM) and the Subtype and Stage Inference (SuStaIn) model to identify and validate distinct ALS subtypes and disease stages; the authors reported dynamic subtype-specific progression patterns and proposed the use of the model for patient stratification and prognosis assessment [134].

Overall, ML has strong potential to integrate multiparametric MRI into clinically useful biomarkers, particularly in DTI-based analyses, supporting more personalized approaches to ALS [18, 135]. However, current models generally perform best in narrowly defined tasks and are most effective as decision-support tools rather than replacements for clinical expertise [136].

### The Future of Imaging in ALS – From Group-Level MRI Findings to Individual Diagnosis

Recent frameworks aim to move from group-level effects to individual diagnosis by mapping disease-specific propagation patterns on single-subject MRI and tracking longitudinal changes in vivo [23, 137]. Achieving reliable clinical application will require dedicated scanning protocols and consistent analysis metrics (e.g., fractional anisotropy for DTI and defined structural measures for T1w imaging), ideally combined with ML approaches. In combination, standardized multiparametric MRI and (AI-based) unbiased analysis could enable accurate, individualized neuroimaging-based diagnostic tools for ALS, but this transition from research biomarkers to clinical read-outs remains an ongoing challenge. Individual prognostic estimation from the neuroimaging-based patterns have been proposed, even in presymptomatic mutation carriers [138] – ethical aspects of a communication of such results at an individual level to a given patient or presymptomatic subject have to be considered in the future, but are beyond the scope of this review.

Future development of neuroimaging biomarkers in ALS requires, as one major challenge, standardized MRI protocols for acquisition parameters and analysis pipelines within large multicenter collaborations, in order to allow translation from group-level findings to reliable individual-level diagnostic and prognostic tools in ALS [19]. While current clinical trials in ALS rely mainly on survival and functional endpoints and fluid biomarkers like neurofilaments have increasingly been investigated [139, 140], neuroimaging mainly with MRI (which has a higher availability than PET) could complement these by providing in vivo mapping in high spatial resolution of regional disease-related progression in the CNS. Because clinical scales like ALSFRS-R incompletely capture cerebral pathology, imaging biomarkers may offer potentially more direct and objective measures of disease progression, also against the background of trial outcomes [18].

In summary, purpose-designed imaging studies have contributed considerable progress over the recent years to analyse MND-related technical fingerprints, to classify individual patients into relevant diagnostic and prognostic categories, and to characterise longitudinal propagation patterns in association with the specific clinical phenotypes. Multiparametric MRI combined with ML shows strong potential to improve sensitivity and to enable individualized patient characterization, outperforming single-modality approaches.

## Key References

- Kleinerova J, Querin G, Pradat PF, Siah WF, Bede P. New developments in imaging in ALS. J Neurol. 2025;272(6):392. https://doi.org/10.1007/s00415-025-13143-8.
  - ○ This study summarizes the departure from describing focal brain changes to focusing on dynamic structural and functional connectivity alterations.
- Lajoie I, Kalra S, Dadar M. Data-driven subtyping and staging of ALS: A multicenter, longitudinal, deformation-based morphometry study. Imaging Neurosci (Camb). 2026;4:IMAG.a.1264. https://doi.org/10.1162/IMAG.a.1264.
  - ○ This longitudinal multicenter study provides a robust model of ALS heterogeneity, showing that subtypes define distinct disease trajectories.
- Müller HP, Kassubek J. Toward diffusion tensor imaging as a biomarker in neurodegenerative diseases: technical considerations to optimize recordings and data processing. Front Hum Neurosci2024;18:1378896. https://doi.org/10.3389/fnhum.2024.1378896.
  - ○ This review summarizes technical considerations for neuroimaging in MND for the optimization of acquisition and analysis protocols.

## Data Availability

No datasets were generated or analysed during the current study.
